# Supplementary material for: Glycosylated SARS-CoV-2 RBD Antigens Expressed in Glycoengineered Yeast Induce Strong Immune Responses Through High Antigen–Alum Adsorption
Source: Biomolecules. 2025 Aug 15;15(8):1172. doi: 10.3390/biom15081172 (PMC12384197; doi:10.3390/biom15081172)
Supplement: Supplementary file 1 [file biomolecules-15-01172-s001.zip › biomolecules-3713016-supplementary.pdf]

# Supplementary Materials

**Table S1.** Oligosaccharide analysis of H-MAN/RBD protein samples.

| Component name | Observed RT (min) | Glycan Units | Observed m/z | Observed mass (Da) | Mass error (ppm) | % Amount (%) |
|---|---|---|---|---|---|---|
| M9 | 23.62 | 9.6485 | 2003.7275 | 2002.7202 | 3.4 | 14.86 |
| M10_iso | 25.73 | 10.4943 | 0 | 0 | 0 | 1.14 |
| M10_iso2 | 25.89 | 10.5628 | 1083.3888 | 2164.7631 | -1.5 | 4.6 |
| M10 | 26.14 | 10.6677 | 1083.3885 | 2164.7625 | -1.7 | 17.1 |
| M10_Phosphorylation | 26.65 | 10.889 | 1123.3717 | 2244.7289 | -1.7 | 10.24 |
| M11_iso | 27.83 | 11.411 | 1164.4123 | 2326.8101 | -3.9 | 1.04 |
| M11 | 28.21 | 11.5839 | 2327.823 | 2326.8157 | -1.4 | 7.29 |
| M11_iso2 | 28.34 | 11.6417 | 0 | 0 | 0 | 3.91 |
| M11_Phosphorylation_iso | 28.62 | 11.7725 | 1204.3987 | 2406.7829 | -1.1 | 2.63 |
| M11_Phosphorylation | 28.83 | 11.8718 | 1204.3992 | 2406.7839 | -0.6 | 8.87 |
| M11_Phosphorylation_iso2 | 29.35 | 12.1186 | 1204.3901 | 2406.7657 | -8.2 | 0.71 |
| M11_Phosphorylation_iso3 | 29.84 | 12.3574 | 1204.3898 | 2406.7651 | -8.5 | 1.09 |
| M12_iso | 30.04 | 12.4561 | 0 | 0 | 0 | 0.74 |
| M12 | 30.27 | 12.5738 | 1245.4327 | 2488.8508 | -8.5 | 3.27 |
| M12_Phosphorylation | 30.68 | 12.7778 | 1285.4192 | 2568.8238 | -5.6 | 7.83 |
| M12_Phosphorylation_iso | 30.91 | 12.8972 | 1285.4187 | 2568.8229 | -6 | 1.13 |
| M12_Phosphorylation2_iso | 31.7 | 13.3123 | 1325.4077 | 2648.8009 | -1.4 | 3.35 |
| M12_Phosphorylation2_iso2 | 32.14 | 13.5549 | 1325.3995 | 2648.7845 | -7.6 | 1.88 |
| M12_Phosphorylation2 | 32.52 | 13.7627 | 1325.4092 | 2648.8037 | -0.3 | 5.03 |
| M13_Phosphorylation2 | 33.44 | 14.2989 | 1406.4355 | 2810.8564 | -0.4 | 3.3 |

| Component name | % Amount (%) |
|---|---|
| Non-Sialylated Glycans | 100 |
| Mono-Sialylated Glycans | 0 |
| Di-Sialylated Glycans | 0 |
| Tri-Sialylated Glycans | 0 |
| De-Fucosylated Glycans | 100 |
| Antennary 1 Glycans | 0 |
| Antennary 2 Glycans | 0 |
| Antennary 3 Glycans | 0 |
| Antennary 4 Glycans | 0 |
| High Mannose Glycans | 100 |

Note: The extended symbol nomenclature for glycans (SNFG) is as follows: A(*) - Antennary (number), with the antennary GlcNAc added to the pentasaccharide core mannose; M# - Mannose (#-3) (number); F(*)# - #(number) fucose 1→*linked fucose; G(*)# - #(number) 1→*linked galactoses; S(*)# - #(number) 2→*linked sialic acids; Lac# - #(number) Galactoses-GlcNAc; Ga: alpha galactoses;MAN- mannose.

**Table S2.** Oligosaccharide analysis of Complex/RBD protein samples.

| Component name | Observed RT (min) | Glycan Units | Observed m/z | Observed mass (Da) | Mass error (ppm) | % Amount (%) |
|---|---|---|---|---|---|---|
| M3B | 8.63 | 4.91 | 1234.4864 | 1233.4791 | 2.6 | 0.53 |
| A1B | 10.46 | 5.47 | 1437.5687 | 1436.5615 | 4.3 | 1.34 |
| M4A1 | 11.69 | 5.83 | 1396.5407 | 1395.5334 | 3.4 | 0.61 |
| M5 | 12.77 | 6.14 | 0 | 0 | 0.0 | 27.56 |
| M4A2 | 13.13 | 6.25 | 1599.6218 | 1598.6145 | 4.0 | 2.28 |
| M4A2_iso | 13.47 | 6.35 | 1599.6243 | 1598.617 | 5.6 | 5.68 |
| M5A1 | 14.40 | 6.62 | 1558.5937 | 1557.5864 | 3.1 | 1.48 |
| M5A2 | 16.09 | 7.11 | 881.3383 | 1760.662 | 0.6 | 54.44 |
| M6A1 | 17.33 | 7.49 | 1720.6497 | 1719.6425 | 4.7 | 3.90 |
| M6A2 | 18.90 | 7.98 | 962.3637 | 1922.7128 | -0.5 | 1.27 |
| M8 | 20.87 | 8.64 | 1841.6664 | 1840.6592 | -0.8 | 0.89 |

| Component name | % Amount (%) |
|---|---|
| Non-Sialylated Glycans | 100 |
| Mono-Sialylated Glycans | 0 |
| Di-Sialylated Glycans | 0 |
| Tri-Sialylated Glycans | 0 |
| De-Fucosylated Glycans | 100 |
| Antennary 1 Glycans | 7.33 |
| Antennary 2 Glycans | 63.67 |
| Antennary 3 Glycans | 0 |
| Antennary 4 Glycans | 0 |
| High Mannose Glycans | 98.13 |

Note: The extended symbol nomenclature for glycans (SNFG) is as follows: A(*) - Antennary (number), with the antennary GlcNAc added to the pentasaccharide core mannose; M# - Mannose (#-3) (number); F(*)# - #(number) fucose 1→*linked fucose; G(*)# - #(number) 1→*linked galactoses; S(*)# - #(number) 2→*linked sialic acids; Lac# - #(number) Galactoses-GlcNAc; Ga: alpha galactoses;MAN- mannose.

**Table S3.** Oligosaccharide analysis of 293F/RBD protein samples.

| Component name | Observed RT (min) | Glycan Units | Observed m/z | Observed mass (Da) | Mass error (ppm) | %Amount (%) |
|---|---|---|---|---|---|---|
| FA1 | 10.23 | 5.36 | 1380.5452 | 1379.5379 | 3.0 | 0.54 |
| FA2 | 12.03 | 5.89 | 1583.6276 | 1582.6203 | 4.5 | 2.97 |
| M5 | 12.91 | 6.14 | 1355.5144 | 1354.5071 | 3.7 | 1.20 |
| FA2B | 13.23 | 6.24 | 893.8537 | 1785.6928 | 0.1 | 3.01 |
| FA2Gn1_iso | 13.73 | 6.38 | 893.8540 | 1785.6935 | 0.5 | 0.49 |
| FA2Gn1 | 13.98 | 6.45 | 893.8531 | 1785.6916 | -0.5 | 1.14 |
| FA3 | 14.33 | 6.55 | 893.8534 | 1785.6922 | -0.2 | 1.91 |
| FA3B | 14.53 | 6.61 | 995.3939 | 1988.7733 | 0.7 | 1.05 |
| FA2G1 | 14.69 | 6.66 | 873.3416 | 1744.6687 | 1.5 | 2.05 |
| FA2G1_iso | 15.04 | 6.76 | 873.3404 | 1744.6663 | 0.2 | 1.14 |
| FA4 | 15.55 | 6.91 | 995.3938 | 1988.7731 | 0.6 | 6.61 |
| FA3G1_iso | 15.91 | 7.01 | 974.8810 | 1947.7475 | 1.1 | 1.30 |
| FA3G1 | 16.46 | 7.18 | 974.8807 | 1947.7469 | 0.8 | 4.65 |
| FA3G1_iso1 | 16.62 | 7.23 | 974.8809 | 1947.7472 | 0.9 | 0.67 |
| FA4G1 | 17.12 | 7.38 | 1076.4201 | 2150.8257 | 0.4 | 2.47 |
| F2A2Gn2_iso | 17.35 | 7.45 | 1068.4219 | 2134.8292 | -0.3 | 1.86 |
| FA2G2 | 17.55 | 7.51 | 954.3677 | 1906.7208 | 1.0 | 2.60 |
| F2A2Gn2 | 17.95 | 7.64 | 1068.4219 | 2134.8293 | -0.3 | 9.29 |
| FA3G2 | 18.09 | 7.68 | 1055.9062 | 2109.7978 | -0.2 | 2.55 |
| FA4G1_iso | 18.33 | 7.75 | 1076.4194 | 2150.8243 | -0.2 | 0.90 |
| A2G2S1_Ac1 | 18.83 | 7.91 | 1047.9090 | 2093.8035 | 17.5 | 6.95 |
| FA3G1S1 | 19.25 | 8.05 | 1120.4265 | 2238.8384 | -1.1 | 7.40 |
| FA2G2S1 | 19.78 | 8.22 | | | | 0.98 |
| FA3G1S1_iso | 20.05 | 8.31 | 1120.4274 | 2238.8402 | -0.3 | 7.31 |
| FA3G1S1_iso1 | 20.49 | 8.46 | 1120.4280 | 2238.8414 | 0.3 | 6.48 |
| FA4G2 | 20.76 | 8.55 | 1157.4472 | 2312.8798 | 1.0 | 0.88 |
| A2G2S2_Ac1 | 21.02 | 8.64 | 1193.4566 | 2384.8987 | 15.2 | 2.91 |
| FA2G2S1_iso | 21.26 | 8.73 | 1099.9131 | 2197.8117 | -1.1 | 3.16 |
| FA3G3 | 21.59 | 8.84 | 1136.9332 | 2271.8519 | 0.4 | 2.86 |
| FA3G2S1 | 22.02 | 9.00 | 1201.4550 | 2400.8954 | 0.8 | 2.08 |
| FA2G1Gn1S2 | 22.29 | 9.10 | 1265.9761 | 2529.9377 | 0.6 | 3.75 |
| A2G2S2_Ac1_iso | 22.77 | 9.27 | 1193.4566 | 2384.8986 | 15.2 | 1.09 |
| FA2G2S2 | 23.43 | 9.52 | 1245.4617 | 2488.9088 | -0.3 | 2.14 |
| FA4G4 | 24.55 | 9.95 | 1319.4979 | 2636.9813 | -0.7 | 0.52 |
| FA3G3S1 | 24.74 | 10.03 | 1282.4808 | 2562.9470 | 0.2 | 0.71 |
| FA3G3S2_iso | 24.90 | 10.09 | | | | 0.33 |
| FA3G3S2 | 25.40 | 10.29 | 1428.0249 | 2854.0352 | -2.3 | 0.31 |
| FA4G2S2 | 25.49 | 10.33 | 1448.5398 | 2895.0650 | -1.2 | 0.30 |
| FA4G4S1 | 26.19 | 10.62 | 1465.0426 | 2928.0706 | -2.8 | 0.90 |
| FA4G4S2 | 27.56 | 11.22 | 1610.5999 | 3219.1853 | 3.5 | 0.55 |

| Component name | % Amount (%) |
|---|---|
| Non-Sialylated Glycans | 52.66 |
| Mono-Sialylated Glycans | 35.97 |
| Di-Sialylated Glycans | 11.38 |
| Tri-Sialylated Glycans | 0.00 |
| De-Fucosylated Glycans | 12.15 |
| Antennary 1 Glycans | 0.54 |
| Antennary 2 Glycans | 41.54 |
| Antennary 3 Glycans | 39.66 |
| Antennary 4 Glycans | 14.18 |
| High Mannose Glycans | 1.20 |

Note: The extended symbol nomenclature for glycans (SNFG) is as follows: A(*) - Antennary (number), with the antennary GlcNAc added to the pentasaccharide core mannose; M# - Mannose (#-3) (number); F(*)# - #(number) fucose 1→*linked fucose; G(*)# - #(number) 1→*linked galactoses; S(*)# - #(number) 2→*linked sialic acids; Lac# - #(number) Galactoses-GlcNAc; Ga: alpha galactoses. Take the glycoform F(6)A4G(4)4S(6)1 as an example: F(6) indicates that there is one fucose, linked in a 1-6 linkage; A4 represents four antennaries; G(4)4 indicates that there are four galactoses linked in a 1-4 linkage; S(6)1 represents one sialic acid linked in a 1-6 linkage.

**Table S4.** Oligosaccharide analysis of L-MAN/RBD protein samples.

| Component name | Observed RT (min) | Glycan Units | Observed m/z | Observed mass (Da) | Mass error (ppm) | % Amount (%) |
|---|---|---|---|---|---|---|
| M8 | 21.22 | 8.7602 | 1841.6753 | 1840.668 | 4 | 3.64 |
| M9 | 23.29 | 9.5197 | 2003.7156 | 2002.7083 | -2.6 | 0.64 |
| M9_iso | 23.69 | 9.6698 | 2003.7305 | 2002.7232 | 4.9 | 7.03 |
| M9_Phosphorylation | 24.41 | 9.952 | 1042.3429 | 2082.6712 | -4.1 | 0.43 |
| M9_Phosphorylation_iso | 24.67 | 10.0587 | 1042.3462 | 2082.6778 | -1 | 5.49 |
| M10 | 25.56 | 10.4202 | 1083.3877 | 2164.7609 | -2.5 | 1.15 |
| M9_Phosphorylation_iso2 | 25.69 | 10.4765 | 1042.346 | 2082.6774 | -1.1 | 1.62 |
| M10_iso | 25.86 | 10.5468 | 1083.3884 | 2164.7622 | -1.9 | 2.83 |
| M10_Phosphorylation | 26.79 | 10.9459 | 1123.371 | 2244.7274 | -2.3 | 2.84 |
| M10_Phosphorylation_iso | 27.01 | 11.0405 | 1123.3731 | 2244.7316 | -0.5 | 2.22 |
| M10__Phosphorylation_iso2 | 27.35 | 11.1886 | 1123.3707 | 2244.7269 | -2.5 | 1.97 |
| M10_Phosphorylation_iso3 | 27.53 | 11.2685 | 1123.3684 | 2244.7223 | -4.6 | 0.91 |
| M11 | 27.9 | 11.4353 | 1164.4091 | 2326.8036 | -6.7 | 0.55 |
| M11_Phosphorylation | 28.62 | 11.7693 | 1204.3975 | 2406.7804 | -2.1 | 0.83 |
| M11_Phosphorylation_iso | 28.66 | 11.7869 | 0 | 0 | 0 | 1.05 |
| M10_Phosphorylation2 | 29.04 | 11.9681 | 1163.3563 | 2324.698 | -0.4 | 17.21 |
| M12 | 29.18 | 12.0351 | 0 | 0 | 0 | 2.11 |
| M12_Phosphorylation | 29.9 | 12.3805 | 1285.4252 | 2568.8359 | -0.9 | 3.63 |
| M11_Phosphorylation2 | 30.15 | 12.5047 | 1244.3832 | 2486.7519 | 0.1 | 10.94 |
| M11_Phosphorylation2_iso | 30.56 | 12.7133 | 0 | 0 | 0 | 1.48 |
| M13 | 30.83 | 12.8508 | 1326.4688 | 2650.9231 | -0.6 | 0.79 |
| M13_iso | 31.19 | 13.0348 | 1326.4624 | 2650.9103 | -5.4 | 1.31 |
| M13_Phosphorylation | 31.43 | 13.1654 | 1366.4494 | 2730.8842 | -2.5 | 5.64 |
| M12_Phosphorylation2_iso | 31.8 | 13.3593 | 1325.4063 | 2648.798 | -2.5 | 0.73 |
| M12_Phosphorylation2 | 32.72 | 13.8728 | 0 | 0 | 0 | 1.14 |
| M13_Phosphorylation2 | 33.17 | 14.1358 | 1406.4361 | 2810.8576 | 0.1 | 10.14 |
| M14_Phosphorylation | 33.96 | 14.6071 | 1487.4619 | 2972.9092 | -0.4 | 11.68 |

| Component name | % Amount (%) |
|---|---|
| Non-Sialylated Glycans | 100 |
| Mono-Sialylated Glycans | 0 |
| Di-Sialylated Glycans | 0 |
| Tri-Sialylated Glycans | 0 |
| De-Fucosylated Glycans | 100 |
| Antennary 1 Glycans | 0 |
| Antennary 2 Glycans | 0 |
| Antennary 3 Glycans | 0 |
| Antennary 4 Glycans | 0 |
| High Mannose Glycans | 100 |

Note: The extended symbol nomenclature for glycans (SNFG) is as follows: A(*) - Antennary (number), with the antennary GlcNAc added to the pentasaccharide core mannose; M# - Mannose (#-3) (number); F(*)# - #(number) fucose 1→*linked fucose; G(*)# - #(number) 1→*linked galactoses; S(*)# - #(number) 2→*linked sialic acids; Lac# - #(number) Galactoses-GlcNAc; Ga: alpha galactoses;MAN- mannose.

**Table S5.** Oligosaccharide analysis of L-MAN-P/RBD protein samples.

| Component name | Observed RT (min) | Glycan Units | Observed m/z | Observed mass (Da) | Mass error (ppm) | % Amount (%) |
|---|---|---|---|---|---|---|
| M5 | 12.79 | 6.14 | 0.0000 | 0.0000 | 0.0 | 34.13 |
| M6 | 15.89 | 7.05 | 1517.5697 | 1516.5625 | 4.9 | 9.27 |
| M7_iso | 18.58 | 7.87 | 840.3106 | 1678.6067 | -0.7 | 0.97 |
| M7 | 18.78 | 7.94 | 1679.6203 | 1678.6130 | 3.1 | 2.65 |
| M8 | 20.85 | 8.63 | 921.3375 | 1840.6605 | -0.1 | 4.89 |
| M8_iso | 21.40 | 8.82 | 1841.6723 | 1840.6650 | 2.4 | 3.90 |
| M8_Phosphorylation | 22.47 | 9.21 | 1921.6396 | 1920.6323 | 2.8 | 2.00 |
| M8_Phosphorylation_iso | 22.92 | 9.38 | 1921.6444 | 1920.6371 | 5.3 | 7.89 |
| M9 | 23.02 | 9.41 | 0.0000 | 0.0000 | 0.0 | 1.95 |
| M9_iso | 23.31 | 9.52 | 1002.3614 | 2002.7082 | -2.6 | 2.88 |
| M9_iso2 | 23.73 | 9.68 | 2003.7140 | 2002.7067 | -3.4 | 0.91 |
| M9_Phosphorylation | 24.50 | 9.99 | 1042.3458 | 2082.6771 | -1.3 | 1.40 |
| M9_Phosphorylation_iso | 24.72 | 10.07 | 1042.3463 | 2082.6781 | -0.8 | 1.29 |
| M9_Phosphorylation_iso2 | 25.12 | 10.24 | 1042.3463 | 2082.6780 | -0.8 | 0.72 |
| M10 | 25.34 | 10.32 | 1083.3890 | 2164.7634 | -1.3 | 4.50 |
| M10_iso | 25.68 | 10.47 | 0.0000 | 0.0000 | 0.0 | 0.41 |
| M10_Phosphorylation | 26.83 | 10.96 | 1123.3713 | 2244.7280 | -2.0 | 0.48 |
| M10_Phosphorylation_iso | 27.02 | 11.04 | 1123.3699 | 2244.7252 | -3.3 | 0.40 |
| M11 | 27.38 | 11.20 | 1164.4125 | 2326.8104 | -3.7 | 1.81 |
| M11_iso | 27.70 | 11.34 | 1164.4154 | 2326.8162 | -1.3 | 2.18 |
| M11_Phosphorylation | 28.31 | 11.62 | 1204.3989 | 2406.7833 | -0.9 | 3.89 |
| M11_Phosphorylation_iso | 28.78 | 11.84 | 1204.3976 | 2406.7806 | -2.0 | 0.39 |
| M11_Phosphorylation_iso2 | 29.09 | 11.99 | 0.0000 | 0.0000 | 0.0 | 2.26 |
| M12_Phosphorylation | 29.89 | 12.37 | 1285.4246 | 2568.8347 | -1.4 | 1.44 |
| M12_Phosphorylation_iso | 30.01 | 12.43 | 0.0000 | 0.0000 | 0.0 | 1.10 |
| M11_Phosphorylation2 | 30.18 | 12.52 | 1244.3822 | 2486.7499 | -0.8 | 1.37 |
| M13_Phosphorylation | 31.47 | 13.18 | 1366.4437 | 2730.8729 | -6.6 | 0.97 |
| M14 | 32.01 | 13.47 | 1407.4943 | 2812.9740 | -1.3 | 0.78 |
| M14_iso | 32.57 | 13.78 | 0.0000 | 0.0000 | 0.0 | 0.41 |
| M13_Phosphorylation2 | 33.20 | 14.15 | 1406.4330 | 2810.8514 | -2.1 | 0.83 |
| M14_Phosphorylation | 33.98 | 14.62 | 1487.4608 | 2972.9071 | -1.1 | 1.92 |

| Component name | % Amount (%) |
|---|---|
| Non-Sialylated Glycans | 100 |
| Mono-Sialylated Glycans | 0 |
| Di-Sialylated Glycans | 0 |
| Tri-Sialylated Glycans | 0 |
| De-Fucosylated Glycans | 100 |
| Antennary 1 Glycans | 0 |
| Antennary 2 Glycans | 0 |
| Antennary 3 Glycans | 0 |
| Antennary 4 Glycans | 0 |
| High Mannose Glycans | 100 |

Note: The extended symbol nomenclature for glycans (SNFG) is as follows: A(*) - Antennary (number), with the antennary GlcNAc added to the pentasaccharide core mannose; M# - Mannose (#-3) (number); F(*)# - #(number) fucose 1→*linked fucose; G(*)# - #(number) 1→*linked galactoses; S(*)# - #(number) 2→*linked sialic acids; Lac# - #(number) Galactoses-GlcNAc; Ga: alpha galactoses;MAN- mannose.

**Table S6.** Zeta Potential of RBD protein samples.

| RBD Protein | Zeta Potential (mV) |
|---|---|
| H-MAN/RBD | −3.672 |
| L-MAN/RBD | −3.688 |
| L-MAN-P/RBD | −1.826 |

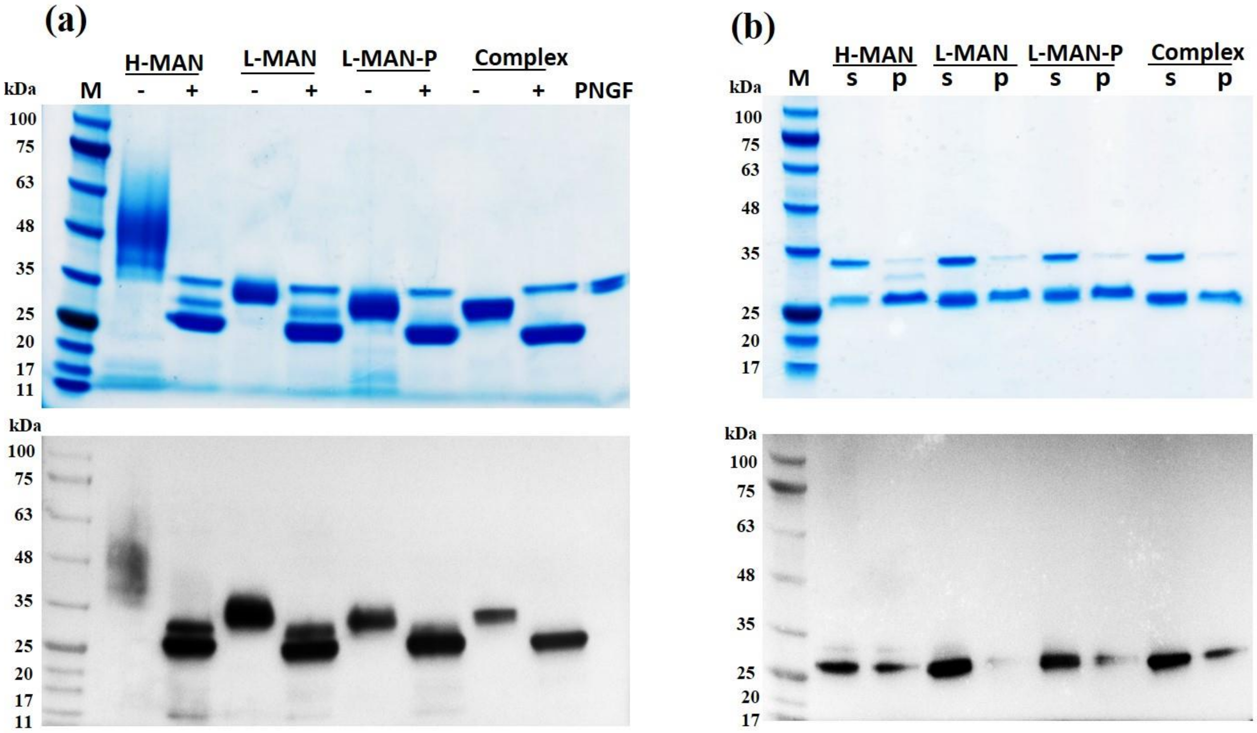


**Figure S1.** The adsorption levels of RBD antigens removed the N-glycans using PNGaseF onto aluminum hydroxide (alum). (**a**) SDS-PAGE and Western blot of RBD antigens with different glycoforms by restriction digestion using PNGF. (**b**) The adsorption supernatant and desorption precipitation of RBD antigens removed the N-glycans using PNGaseF onto aluminum hydroxide.
